# Evaluating Patient-Entered Electronic Health Data as a Strategy to Improve Quality of Care in a Diabetes Clinic: Protocol for a Randomized Controlled Trial

**DOI:** 10.2196/89519

**Published:** 2026-05-08

**Authors:** Julia Hunter-Schouela, Lindsey Ure, Caroline Zuijdwijk, Elias Abou-Assaly, Alexandra Ahmet, Jennifer de Boer, Karine Khatchadourian, Stephen A Kutcher, Sarah E Lawrence, Deepti Reddy, Richard Webster, Ellen B Goldbloom

**Affiliations:** 1 Division of Endocrinology, Children's Hospital of Eastern Ontario, 401 Smyth Road, Ottawa, ON, K1H 8L1, Canada, 1 613-737-7600; 2 Faculty of Medicine, University of Ottawa, Ottawa, ON, Canada; 3 Children's Hospital of Eastern Ontario Research Institute, Children's Hospital of Eastern Ontario, Ottawa, ON, Canada; 4 eHealth and Information Services, Children's Hospital of Eastern Ontario, Ottawa, ON, Canada; 5 Telfer School of Management, University of Ottawa, Ottawa, ON, Canada

**Keywords:** type 1 diabetes, digital health, informatics, patient portal, electronic health record, quality of care, learning health system, randomized controlled trial

## Abstract

**Background:**

Quality care for pediatric type 1 diabetes (T1D) requires frequent, multidisciplinary visits. Technological and clinical innovation have led to changes in T1D management, resulting in increasing data exchange required during these visits. Capturing comprehensive personal health and diabetes-related information discretely and integrating it into the clinical workflow is critical for optimal T1D care but is time-consuming. Time spent on data transfer often results in less time for holistic care and can lead to unmet needs for patients, families, and health care providers, as well as increased time pressures in clinic. To address this, the Children’s Hospital of Eastern Ontario developed a caregiver proxy-reported questionnaire distributed via the MyChart patient portal, allowing families to input care information ahead of visits with the aim of dedicating more clinic time to personalized care. The launch of this tool, which integrates caregiver-entered information into the physician’s documentation workflow, brings the opportunity to systematically evaluate its impact on care quality and efficiency, with potential implications for broader adoption.

**Objective:**

Our objective is to evaluate the impact of a caregiver proxy-reported, electronic health record–integrated preclinic questionnaire (MyChart questionnaire) on the quality of care in a pediatric diabetes clinic, through measurement of its impact on caregiver-perceived quality of care compared to the standard of care using 2 validated measures of care quality. We also aim to explore the impact of the intervention on glycemic control and visit efficiency.

**Methods:**

We conducted a single-center, parallel-group randomized controlled trial designed for 222 children with T1D. Participants were randomly allocated in a 1:1 ratio to either the intervention (MyChart questionnaire) or standard care. Our primary outcome is caregiver-perceived quality of care, as measured by the Patient’s Evaluation of the Quality of Diabetes Care at 8 months, administered with caregivers serving as proxy respondents for patients. Secondary outcomes are the Patient’s Evaluation of the Quality of Diabetes Care at 4 months and Perceived Quality of Medical Care at 4 and 8 months. Tertiary outcomes include glycemic control and physician-reported visit efficiency at 4 and 8 months. Analysis of covariance models will be used to assess changes between baseline and postintervention outcomes across treatment groups.

**Results:**

Recruitment for this study began in April 2023 and was completed in February 2024, with a total of 139 participants enrolled. Data collection has concluded, and the first results are expected in the spring of 2026.

**Conclusions:**

This study is the first randomized trial to assess the impact of a caregiver proxy-reported, electronic health record–integrated, preclinic questionnaire distributed via a patient portal on caregiver-perceived quality of care in a pediatric care setting. The results will guide changes in health service infrastructure and delivery to enhance comprehensive data capture and improve care quality within and beyond pediatric T1D.

## Introduction

### Background and Rationale

According to the World Health Organization (WHO), quality health care must be safe, effective, timely, efficient, equitable, integrated, and people-centered [1]. In this regard, international guidelines [2] recommend that optimal type 1 diabetes (T1D) care delivery involves 4 visits per year with a multidisciplinary team. Advancing technology and clinical innovation have led to substantial changes in T1D management, requiring the collection of a greater breadth and depth of information from patients, as well as more data to review during each visit. While expected to improve patient care, these changes have resulted in a larger proportion of the physician-patient visit being dedicated to data collection and entry at the expense of actual T1D care [3], including psychosocial aspects. This has led to unmet needs for patients, families, and health care providers alike, while contributing to increased time and space pressures in clinics.

**Figure 1.**
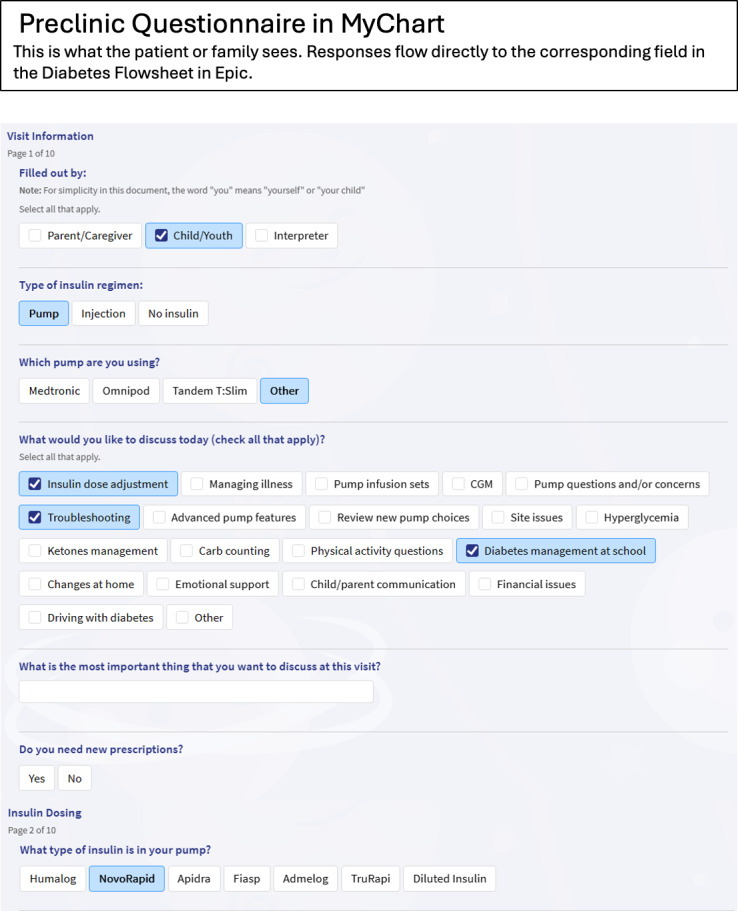
Screen capture of sample questions in the MyChart questionnaire (Copyright ©2026 Epic Systems Corporation).

**Figure 2.**
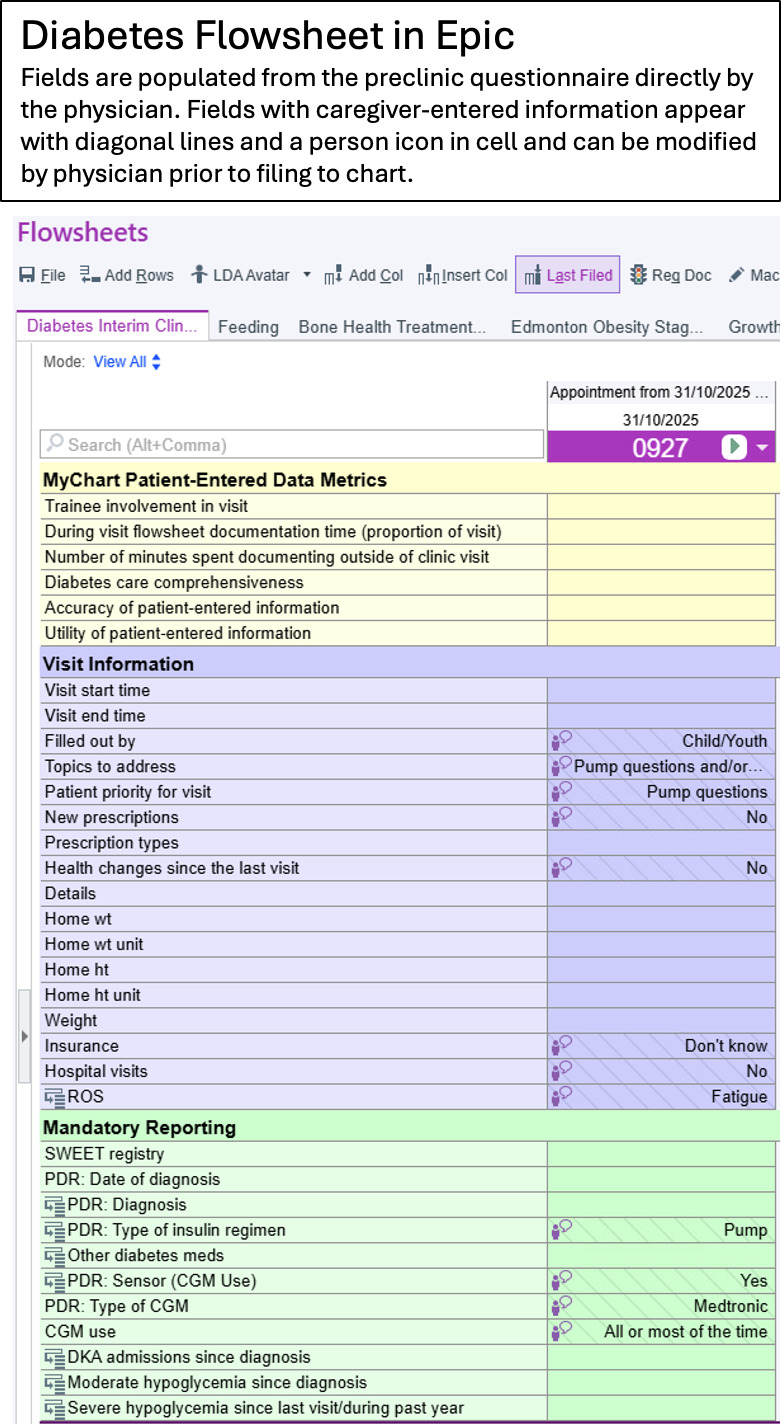
Screen capture of a portion of the Diabetes Flowsheet physician documentation tool. Information from the MyChart questionnaire (Epic) flows directly to the Diabetes Flowsheet and appears as patient-entered, allowing for modification before filing to the chart and directly populating templated physician clinic notes (Copyright ©2026 Epic Systems Corporation).

One way to increase both efficiency and quality of care during a visit is to allow patients and families to provide information in advance. While preclinic questionnaires are common, having the information incorporated directly into the physician’s documentation is not. Epic is the most widely used electronic health record (EHR) in the United States, with growing uptake in Canada [4], including use at the Children’s Hospital of Eastern Ontario (CHEO). An EHR-integrated caregiver proxy-reported questionnaire using CHEO’s secure patient portal, Epic MyChart, has recently been developed through a partnership between CHEO’s diabetes and eHealth teams (Figure 1). Prior to a T1D clinic visit, the families use the MyChart questionnaire to enter details about current management and visit goals, which automatically populate corresponding fields within their EHR for subsequent review by the health care team (Figure 2), before and during the visit, with the goal of reallocating clinic time previously used for data acquisition to individual patient priorities (wise resourcing). The tool and workflow underwent qualitative piloting with 7 patients and 3 providers in our clinic, demonstrating feasibility and acceptability prior to implementation. At our center, existing infrastructure allows for the incorporation of the preclinic MyChart questionnaire through a built and vetted diabetes flowsheet that is already the central document for recording discrete, comprehensive clinical information. Since its inception 9 years ago, the flowsheet has been customized and optimized to suit the needs of this population. The CHEO Diabetes Flowsheet has been shared with pediatric centers across Canada and has informed the development of a tool created by the International Epic Pediatric Endocrinology Specialty Steering Board, which is available in the Epic EHR foundation system (available to all Epic clients). This flowsheet is part of a standard workflow adopted by all CHEO-based physicians providing diabetes care. Capturing discrete data within this comprehensive flowsheet allows for easy access to historical and current clinical information during and between visits, as well as for tracking over time. This functionality sets the stage for easier patient-entered and provider-entered data capture, storage, reporting, and database development, which are critical for quality assurance and research endeavors.

Studies have shown that the accuracy of patient-entered information related to specific conditions can be high [5,6], and that the use of questionnaires outside of the clinical visit via a patient portal can be an effective means of obtaining relevant patient information prior to the clinic visit [7]. Patient and caregiver-facing technologies allowing direct EHR-data entry in certain care settings have provided new, meaningful additions to the clinical record; however, there is a paucity of evidence systematically evaluating their impact. Formal assessment of the impact of EHR tools is essential to assess their impact on the quality of care, usability, and health outcomes [8-11].

Our goal is to improve the quality of care in the CHEO T1D clinic. Patient experience is a critical element for sustained change in any care model [12,13]. Patient-reported experience measures (PREMs)—questionnaires that capture patients’ perceptions of their care—are increasingly recognized as key indicators of care quality and were used in this study [14,15]. In pediatric contexts, care experiences are often captured through caregiver reporting [16,17], as was the case in our study.

Capturing comprehensive personal health and diabetes-related information discretely and integrating it into the clinical workflow is critical for the optimal care of individuals with T1D. With the current workflow, it is not feasible for providers to collect all the data required while thoroughly addressing diabetes care issues during a single clinic visit. In response, we have developed an electronic preclinic MyChart questionnaire to collect and directly integrate data into the EHR and clinical workflow. The launch of the MyChart questionnaire brought the opportunity to systematically evaluate this method of data collection and care delivery. Without evaluation, this model could alter T1D clinic workflows without providing benefit—and could even risk diminishing visit quality. Conversely, evaluation offers the opportunity to demonstrate the value of an EHR-integrated preclinic MyChart questionnaire, with the potential to inform and improve care delivery in other pediatric T1D clinics and beyond.

### Objectives

The main objective of this study is to assess the effect of an EHR-integrated caregiver proxy-reported preclinic questionnaire on postappointment caregiver-perceived quality of care in a T1D clinic. Other objectives include evaluating the impact of the MyChart questionnaire on glycemic control and visit efficiency. Finally, exploratory objectives focus on caregiver uptake of the tool, as well as caregiver and provider feedback about the tool and associated workflow to inform future implementation.

### Hypotheses

We hypothesize that the use of an EHR-integrated caregiver proxy-reported preclinic MyChart questionnaire in the CHEO T1D clinic will improve caregiver-perceived quality of care.

## Methods

### Ethical Considerations

This study was reviewed and approved by the local research ethics board at CHEO (protocol 22/99X, approved September 1, 2022). Study participants were recruited in accordance with the Personal Health Information Protection Act, using both in-clinic and out-of-clinic strategies. After being introduced by a member of the participant’s circle of care, a research team member obtained written informed consent—either in person or remotely—using the REDCap (Research Electronic Data Capture; Vanderbilt University) system [18,19].

Consent was obtained either virtually via REDCap, verbally by telephone, or in person. Capacity to consent or assent was determined by a delegated member of the participant’s health care team. However, as a general guideline, participants aged less than 11 years were asked for assent, and a consent form was provided to the child’s caregiver. Given that all participants were aged 11 years or younger, we anticipated that no child participant had the capacity to consent on their own behalf, meaning that parents or caregivers participated on their behalf (and that caregivers completed the study procedures and questionnaires). Additionally, the choice to include participants aged less than 11 years was made in order to select a patient population where all caregivers would be able to obtain MyChart access. Caregivers at CHEO were defaulted to act as their child’s proxy via MyChart until each patient reached the age of 12 years, at which time MyChart access was no longer extended to caregivers without the patient’s explicit consent (unless the patient lacked capacity to consent). In light of this, the initial participant age of less than 11 years allowed for patients to remain under 12 years for the duration of the study. Participants who took part in the study received a CAD $10 (1 CAD=US $0.71) Amazon gift card for each study visit completed, for a maximum of CAD $30.

### Study Design

We conducted a single-center, parallel-group randomized controlled trial assessing the effect of a patient-facing EHR-integrated preclinic MyChart questionnaire (intervention) on caregiver-perceived quality of care in patients with T1D followed over 3 diabetes clinic visits (1 baseline visit and 2 follow-up visits), each approximately 4 months apart, compared with the control group (no preclinic MyChart questionnaire or standard of care).

### Participants and Recruitment

Children with T1D, aged less than 11 years, were recruited from the T1D Clinic at CHEO in Ottawa, Ontario, Canada. Potential participants were identified in one of the following ways: (1) an Our Practice Advisory (previously known as Best Practice Advisory), programmed by a data warehouse team to automatically send an in-basket message in the EHR to the study coordinator for patients meeting inclusion criteria, or (2) by manual screening of diabetes clinic schedules in the Epic EHR system by the study coordinator.

### Inclusion and Exclusion Criteria

Children who met eligibility criteria were invited to participate in the study. Children aged less than 11 years with a T1D diagnosis were approached for informed consent. As outlined in Textbox 1, patients were excluded if they were not fluent in English, were actively being followed in an eating disorder clinic or a Child and Youth Protection clinic, or were unable to provide consent.

Textbox 1. **Inclusion and exclusion criteria.**

**Inclusion criteria**

- Diagnosis of type 1 diabetes
- Aged less than 11 years at enrollment

**Exclusion criteria**

- Not fluent enough in English to complete all study-related procedures
- Unable or unwilling to provide consent and/or assent
- Followed in eating disorder clinic or Child and Youth Protection Clinic (there are patient-facing electronic health record restrictions for individuals followed in these clinics)

### Trial Intervention

**Figure 3.**
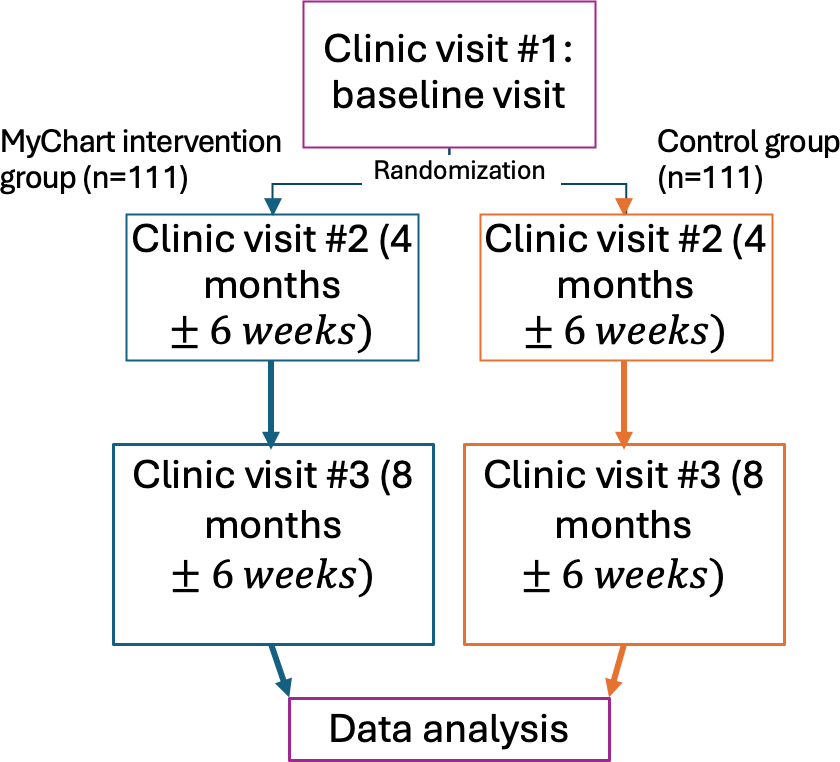
Schematic overview of the study.

As shown in Figure 3, participants from the T1D clinic at CHEO were randomized to the MyChart questionnaire intervention group or the control group (standard of care).

### Intervention Group  

The MyChart intervention group was invited to complete the MyChart questionnaire up to 7 days prior to clinic visits 2 and 3. A reminder was also sent the day prior to their clinic visit if they had not yet completed the questionnaire. The MyChart questionnaire allows questionnaire answers to be submitted through the secure patient portal and populate the Diabetes Flowsheet in the patient’s EHR. The questionnaire collects patient-reported clinical information, such as health changes, current management, and clinic visit goals. The complete questionnaire contents can be seen in Multimedia Appendix 1. A visual sample of the questionnaire as it appeared to study participants is shown in Figure 1.

### Control Group

Participants in the control group received standard-of-care treatment as usual, with no preclinic MyChart questionnaire.

### Randomization Procedures

Once informed consent was obtained, participants were randomly assigned in a 1:1 ratio to either the intervention group or standard care (no preclinic MyChart questionnaire). A statistician, not directly involved in recruitment, generated the block randomization list which was uploaded to REDCap. Randomization was conducted by the study coordinator using the randomization module in REDCap, blinding the study team to the allocation sequence.

### Data and Outcome Measures

Demographic, clinical, and administrative health care data for baseline characteristics, subanalyses, and applicable outcome measures were collected from the EHR using a customized report, chart review, and case report form. Case report forms used at baseline and follow-up can be seen in MultimediaAppendices 2  3.

We collected the following demographic information at baseline: age at study start, sex, date of diabetes diagnosis (to enable calculation of diabetes duration), glycated hemoglobin (HbA_1c_) levels over the preceding year (to compute mean HbA_1c_ over the year), recorded Glucose Management Index over the preceding year (to compute mean Glucose Management Index over the year), blood glucose percent time in range as measured by continuous glucose monitoring (CGM) at baseline, number of missed appointments (no-shows) with their diabetes physician in the preceding year, insulin regimen (pump or injection), and CGM use (yes or no).

We collected the following patient characteristics at each study visit: age, insulin regimen, CGM use (yes or no), visit type (in-person vs virtual), and documenting provider (staff vs trainee).

In order to assess the effect of the MyChart questionnaire on caregiver-perceived quality of care, caregivers were invited to complete 2 validated PREMs at baseline and during the 2 follow-up study visits, at 4 and 8 months: Patients’ Evaluation of the Quality of Diabetes Care (PEQD), a 14-item scale [20], and Perceived Quality of Medical Care (PQMC), a 6-item instrument [21].

The primary outcome for this study is the 14-item PEQD scale at 8 months. Secondary outcomes include the PEQD at 4 months and the PQMC instrument, measured at both 8 and 4 months.

Tertiary outcomes include glycemic control, as measured by HbA_1c_ at each visit, and visit efficiency, as measured through the provider-reported estimate of their data collection time for both groups (see “during visit flowsheet documentation time row” in Figure 2). Options for provider-reported “during visit flowsheet documentation time” included the following proportions: less than 10%, 10% to 30%, 31% to 50%, 51% to 70%, and greater than 70%. A sensitivity analysis for glycemic control using the blood glucose percent time in range at each visit is planned.

To understand our exploratory outcomes and the implementation impact of the novel intervention and workflow (for the intervention group only), and to inform continuous quality improvement, patients and providers were surveyed. Survey tools included: (1) an end-of-study patient survey about the preclinic questionnaire and study visit clinic workflow, (2) an end-of-study provider survey, and (3) provider-perceived efficiency or accuracy measures collected at each visit. MyChart activation and MyChart questionnaire completion rates will also be assessed. The end of study participant and provider surveys can be seen in MultimediaAppendices 4  5.

### Trial Management

**Figure 4.**
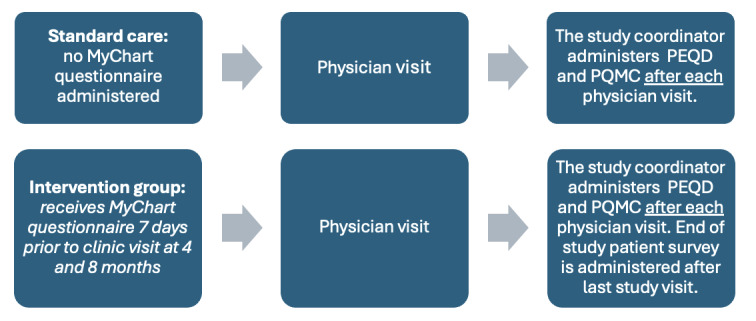
Schematic depiction of participant experience during study visits. PEQD: Patient’s Evaluation of the Quality of Diabetes Care; PQMC: Perceived Quality of Medical Care.

To facilitate patient portal (MyChart) enrollment (required for the completion of the MyChart questionnaire) for participants not already active on the portal, the study coordinator was trained to provide instant activation by CHEO’s Health Information Management and MyChart team (Figure 4).

At each study visit, the study coordinator extracted clinical information outlined in Table 1 from the patient chart and continuous glucose monitor portal (shared by patients with the clinic as part of routine clinical care), using a study case report form, and had the participant complete the associated study questionnaires via REDCap.

Following the completion of data collection, the providers were asked to complete a survey. All CHEO physicians who provide care in the diabetes clinic acted as providers in this study, as part of their standard clinical care. These physicians were involved in the development of the protocol and agreed to be involved in this capacity. If providers decided not to participate in this capacity, the transition of care to another CHEO diabetes physician was to be offered.

**Table 1.** Data collected and surveys completed during study clinic visits.

	Method of data retrieval	Baseline assessment	4-month visit (±6 wk)	8-month visit (±6 wk)
Baseline demographic and medical history	Obtained from EHR^a^^b^	✓		
Visit-level medical information	Obtained from EHR^b^	✓	✓	✓
MyChart Questionnaire (Epic) intervention group only, sent 7 days prior to 4 and 8 month clinic visits	Completed via MyChart		✓	✓
PQMC^c^ (6-item)	Completed via REDCap^d^	✓	✓	✓
PEQD^e^ (14-item)	Completed via REDCap	✓	✓	✓
Participant end-of-study survey	Completed via REDCap			✓
Glycemic measures	Obtained from shared continuous glucose monitor platforms and EHR	✓	✓	✓
Efficiency measures	Documented in EHR	✓	✓	✓
Provider end-of-study survey	Completed via REDCap			✓

a EHR: electronic health record.

b REDCap case report forms were used to capture data manually extracted from EHR.

c PQMC: Perceived Quality of Medical Care.

d REDCap: Research Electronic Data Capture.

e PEQD: Patient’s Evaluation of the Quality of Diabetes Care.

### Equity, Diversity, and Inclusion Considerations

Our T1D demographic is diverse in ethnicity, gender, and socioeconomic status [22]. For this study, one limitation was that only patients fluent in English were eligible; this includes the majority, but not all, of our clinic population. This decision was made due to feasibility constraints in obtaining validated, usability-tested translations in multiple languages for the purposes of this initial investigation. Favorable results will serve as impetus for expanding this tool for use by non–English-speaking families.

### Statistical Plan

#### Sample Size

The sample size was estimated using the *power_oneway_ancova*() function from the *Superpower* package in R (R Foundation for Statistical Computing) [23]. Assuming a baseline PEQD patient satisfaction score of 80/100, a SD of 20.8 ([range/4+range/6] ÷ 2; modified from Hozo et al [24]), an equal 1:1 random allocation per study arm, and a 5% type-I error rate, a sample size of 94 participants per treatment group would provide 90% power to detect a 10% absolute between-group improvement in patient satisfaction. Furthermore, assuming 15% of participants are lost to follow-up, the required sample size was inflated to 111 per group, for a total sample size of 222.

#### Analysis for Primary Outcomes

Using the intention-to-treat principle, the primary analysis will compare the post-intervention outcome between treatment groups using analysis of covariance (ANCOVA). The ANCOVA model will include the postintervention PEQD score at 8 months as the dependent variable, the treatment group as the primary independent variable, and the baseline value of the PEQD score as a covariate to account for individual differences at baseline.

#### Analyses for Secondary Outcomes

The secondary analyses will include 3 ANCOVA models. The first will include the PEQD score at 4 months as the dependent variable with subsequent modeling of the postintervention PQMC score at 8 months and 4 months as the dependent variables.

#### Analyses for Tertiary Outcomes

Tertiary analyses using ANCOVA modeling will compare preintervention and postintervention glycemic control, as measured by HbA_1c_ as the dependent variable, at 8 months and then 4 months between treatment groups. A sensitivity analysis will be performed using blood glucose percent time in range. Furthermore, visit efficiency, as measured by the provider-reported proportion of time spent on documentation during visits at baseline and 8 months, will be compared between treatment groups.

#### Analysis for Exploratory Outcomes

The exploratory objective analysis will focus on the intervention arm. It will include an assessment of patient uptake of the tool (MyChart activation and questionnaire completion rates) as well as patient and provider feedback about the tool and associated workflow (obtained via surveys), using descriptive statistics.

#### Multiple Testing

To minimize concerns regarding multiplicity, a Holm correction, which penalizes each additional statistical test, will be applied to all reported *P* values across the secondary and tertiary outcomes. For exploratory outcomes, their proportions and 95% CIs will be reported, where appropriate.

#### Missing Data

Any missing data for the primary outcome, PEQD, will be addressed using multiple imputation. The imputation model will incorporate both baseline and post-randomization outcome variables to ensure valid inferences under the assumption that data are missing at random. Assessments of the variable distributions, such as linearity and skewness, will be conducted to inform the selection of the multiple imputation approach. There will be no planned imputation of any secondary or tertiary outcomes.

#### Statistical Programming

All analyses will be completed using the R statistical programming software (R Foundation for Statistical Computing) [25], and Subversion (Apache Software Foundation) version control will be used to ensure reproducibility and code integrity through the tracking of code changes.

## Results

Study recruitment began in April 2023 and was completed in February 2024, with 139 participants successfully enrolled. Data collection concluded on May 26, 2025, and results are expected in the spring of 2026.

The 139 recruited participants are fewer than our initial sample size of 222 and will influence our power to detect an improvement in patient satisfaction. The impact of the smaller recruited sample size will lower our statistical power from 90% to 80% to detect a 10-point difference, with adjustment of our initial assumption from the 15% expected loss to follow-up to instead having no expected loss-to-follow-up, with the imputation of missing outcome values. Another perspective is that we would retain 90% power to detect a 10-point difference in the outcome if we adjust our SD estimate (range/6 = 16.7) to reflect those recommended by Hozo et al [24] for larger sample sizes (n>70). Even with lower recruitment numbers, we anticipate our evaluation to remain well-powered (>80%) to detect a 10-point improvement in patient satisfaction.

## Discussion

### Expected Findings

In this study, we will evaluate the use of a patient-facing, EHR-integrated pre-clinic MyChart questionnaire in a pediatric T1D clinic as a means to improve the quality of care. We will also assess the impact of this intervention on glycemic outcomes and visit efficiency. We anticipate that our customized, intuitive patient-facing interface will increase data capture and free up time during the visit to identify and focus on priority issues, helping providers better and more efficiently support their patients. We hypothesize that this will translate into improved PREMs at 4 and 8 months in patients completing these questionnaires as compared to a control group.

Demonstrating a favorable impact of this tool and workflow would provide a rationale for this infrastructure design to be sustained in our clinic and shared with other diabetes clinics. The design can be applied to create similar EHR workflows that accommodate patient-entered data, including PREMs, into clinical documentation and care across a broad range of conditions.

### Strengths and Limitations

To our knowledge, this study will be the first randomized controlled trial to assess how caregiver-perceived quality of care is impacted by the use of a caregiver proxy-reported, EHR-integrated, preclinic questionnaire distributed via a patient portal. Methodologically rigorous research in this area is lacking, and there is a need for this to be completed and reported in sufficient detail to allow for future replication and subsequent generalizability [26-28]. Evaluation of how a patient-facing interface customized to existing workflows can help providers better and more efficiently support their patients is required to justify changes in health service infrastructure and delivery. Furthermore, patient-entered information should enhance data that exist in patients’ medical records—which, when combined and analyzed, can help to better understand differences in patient care and subsequently improve patient care and outcomes [29].

Incorporation and integration of increasingly complex patient data, personal health information, and diabetes-related information into the clinical workflow are essential for understanding our patients and optimizing care. It nonetheless poses a challenge to our resource-constrained health care system and is currently not done effectively [30]. Partnering with families to allow them to provide their own information prior to a visit, directly into their EHR in a discrete, efficient, and interpretable manner, provides an innovative way to address this challenge. Additionally, this study prioritizes the patient experience as the primary outcome, recognizing that it is a critical element in any sustained change in care models and health outcomes [12,13].

Feasibility and acceptability are important considerations for any clinical intervention. The collection of MyChart survey completion rates, as well as surveying both patients and providers involved in diabetes care, will enable further assessment of feasibility and acceptability, respectively.

There are some identified limitations to this study. The trial is not blinded, as participants, care providers, and the data analysis team could not be blinded to participant assignment groups. We also recognize the inclusion of only English-speaking participants, representing at least 85% of our patient population, and those aged under 11 years at enrollment as limitations. At our center, youth aged 12 years or older begin to control access to their patient-facing EHR account, which adds complexity related to portal use in adolescence. If favorable results ensue from this work, further study would expand the use of this tool in older age groups and address language and literacy barriers. Planned mitigation strategies for more widespread and equitable access include formal translation into the most commonly spoken non-English languages in our clinical population, with professional translation services to ensure linguistic appropriateness. We also plan to conduct language-specific usability and readability testing prior to broader implementation. Caregiver feedback provided in this study will also be used to further optimize future iterations. Furthermore, with respect to generalizability, we note that the use of electronic means of administering a patient-facing questionnaire may be a barrier [31]. Measures such as tablet access in the waiting room in advance of patient clinic appointments should be considered to optimize inclusivity. Continued efforts to ensure that electronic means of health care provision are readily accessible are paramount to avoid further exacerbating existing care disparities.

### Conclusions

If the use of a patient-facing, EHR-integrated preclinic MyChart questionnaire in a pediatric T1D clinic is shown to be effective, it will help inform the reorganization of care delivery and has the potential to impact patient outcomes beyond our institution and diabetes-specific care. It will also provide the necessary infrastructure to address longer-term diabetes-related health outcomes, to answer important quality improvement and research questions, and to increase data capture for provincial and national diabetes registries currently under development.

A diabetes flowsheet developed at our center has been adopted at a number of other Canadian pediatric centers, and if proven efficacious, we anticipate that this tool will be adopted at these other sites, further facilitating multicenter collaboration with respect to both health care delivery and diabetes research.

We are living in historic times captured by the dramatic ascendancy of diabetes technologies, virtual care, and the use of artificial intelligence in the medical realm. Diabetes is ideally suited for this new era of medicine that leverages digital solutions to improve patient care. The infrastructure built and evaluated in this study can be expanded to other populations (broadening age, language, accessibility, and conditions) and can be applied to create similar disease-specific EHR workflows that accommodate patient-entered data, including patient-reported outcome and experience measures, into clinical documentation and care provided virtually or in person. This innovation will ease our continued transition to this new virtual era across a broad range of conditions and provide a much-anticipated solution for the incorporation of PREMs into routine care.

## Supplementary material

10.2196/89519 Multimedia Appendix 1 MyChart questionnaire contents. A visual representation of how this appeared to participants is included in Figure 1.

10.2196/89519 Multimedia Appendix 2 Baseline demographic and medical history form.

10.2196/89519 Multimedia Appendix 3 Visit level medical information form.

10.2196/89519 Multimedia Appendix 4 Participant end of study survey.

10.2196/89519 Multimedia Appendix 5 Provider end of study survey.

10.2196/89519 Peer Review Report 1 Peer review report by Children's Hospital Academic Medical Organization (CHAMO) Innovation Fund, Children’s Hospital of Eastern Ontario (CHEO) Research Institute (Canada).

## Abbreviations

ANCOVA: analysis of covariance
CGM: continuous glucose monitoring
CHEO: Children’s Hospital of Eastern Ontario
EHR: electronic health record
HbA_1c_: glycated hemoglobin
PEQD: Patients’ Evaluation of the Quality of Diabetes Care
PQMC: Perceived Quality of Medical Care
PREM: patient-reported experience measure
REDCap: Research Electronic Data Capture
T1D: type 1 diabetes
WHO: World Health Organization
