# Supplementary material for: Evaluating Patient-Entered Electronic Health Data as a Strategy to Improve Quality of Care in a Diabetes Clinic: Protocol for a Randomized Controlled Trial
Source: JMIR Res Protoc. 2026 May 8;15:e89519. doi: 10.2196/89519 (PMC13155505; doi:10.2196/89519)
Supplement: Multimedia Appendix 1 [file resprot-v15-e89519-s001.docx]

**MyChart Patient Questionnaire**

Table of Contents

## [Visit Information](#Visitinfo)

## [Insulin Dosing](#InsulinDose) and [Injection Dose](#InjectionDose)

## [Insulin Delivery](#InsulinDelivery)

## [Blood Sugar Monitoring](#BSMonitoring)

## [High BG and Ketones](#BGandKetones)

## [Hypoglycemia](#Hypoglycemia1)

## [Nutrition](#Nutrition1) and [Physical Activity](#PhysicalActivity)

## [Other](#Other1)

## [Your Health](#Yourhealth)

**Visit Information**

1. Filled out by

Note: For simplicity in this document, the word “you” means “yourself” or “your child”

Select all that apply:

- Parent/Caregiver
- Child/Youth
- Interpreter

1. Type of insulin regimen:

- Pump
- Injection
- No insulin

1. Which pump are you using?

- Medtronic
- Ominpod
- Tandem
- Ypsomed
- Other

1. What would you like to discuss today?

Select all that apply:

- Insulin dose adjustment
- Managing illness
- Pump infusion sets
- CGM/FGM
- Pump questions and/or concerns
- Troubleshooting
- Advanced pump features
- Review new pump choices
- Site issues
- Hyperglycemia
- Ketones management
- Carb counting
- Physical activity questions
- Diabetes management at school
- Changes at home
- Emotional support
- Child/parent communication
- Financial issues
- Driving with diabetes
- Other

1. What is the most important thing that you want to discuss at this visit?
2. Do you need new prescriptions?

- Yes
- No

1. What for?

Select all that apply:

- Insulin
- Glucagon
- Diabetes supplies

1. Have there been any changes to your overall health or medications (other than insulin) since the last visit?

- Yes
- No

1. What are the changes?
2. Have you had any hospital visits (emergency room and/or admission) since your last visit?

- Yes
- No

**Insulin Dosing**

1. What type of insulin is in your pump?

- Humalog
- NovoRapid
- Apidra
- Flasp
- Admelog
- TruRapi
- Diluted Insulin

1. When are meal and snack boluses given?

Select all that apply:

- More than 5 minutes before
- Just before eating
- During meal
- After eating

1. More than 5 minutes before:
   - Always
   - Often
   - Sometimes
   - Rarely
2. Before eating:
   - Always
   - Often
   - Sometimes
   - Rarely
3. During a meal:
   - Always
   - Often
   - Sometimes
   - Rarely
4. After a meal:
   - Always
   - Often
   - Sometimes
   - Rarely

**Injection Dose**

1. How do you give/receive insulin injections?

Select all that apply:

- Syringes
- Pens

1. What type of Basal (long-acting) insulin do you use?

- Humulin N
- Novolin NPH
- Levemir
- Lantus
- Basaglar
- Toujeo
- Tresiba
- Other
- None

1. When do you usually take your Humulin N?

- Morning
- Midday
- Afternoon
- Suppertime
- Bedtime
- Other

1. When do you usually take your Novolin NPH?

- Morning
- Midday
- Afternoon
- Suppertime
- Bedtime
- Other

1. When do you usually take your Levemir?

- Morning
- Midday
- Afternoon
- Suppertime
- Bedtime
- Other

1. When do you usually take your Lantus?

- Morning
- Midday
- Afternoon
- Suppertime
- Bedtime
- Other

1. When do you usually take your Basaglar?

- Morning
- Midday
- Afternoon
- Suppertime
- Bedtime
- Other

1. When do you usually take your Tresiba?

- Morning
- Midday
- Afternoon
- Suppertime
- Bedtime
- Other

1. When do you usually take your Toujeo?

- Morning
- Midday
- Afternoon
- Suppertime
- Bedtime
- Other

1. When do you usually take your other insulin?

- Morning
- Midday
- Afternoon
- Suppertime
- Bedtime
- Other

1. MORNING – What would be a “typical” dose when your blood sugar is between 4 and 8 mmol/L?
2. MIDDAY – What would be a “typical” dose when your blood sugar is between 4 and 8 mmol/L?
3. AFTERNOON – What would be a “typical” dose when your blood sugar is between 4 and 8 mmol/L?
4. SUPPERTIME – What would be a “typical” dose when your blood sugar is between 4 and 8 mmol/L?
5. BEDTIME – What would be a “typical” dose when your blood sugar is between 4 and 8 mmol/L?
6. OTHER – What would be a “typical” dose when your blood sugar is between 4 and 8 mmol/L?
7. What type of rapid (fast acting) insulin do you use?

Select all that apply:

- Humalog
- NovoRapid
- Apidra
- Flasp
- Admelog
- TruRapi
- Diluted Humalog
- Entuzity
- Other

1. When do you usually take your rapid (fast acting) insulin?

- Breakfast
- Morning snack
- Lunch
- Afternoon snack
- Dinner
- Bedtime snack
- Overnight
- Other

1. BREAKFAST- Do you use a ratio or fixed/set dose?

- Ratio
- Fixed/Set dose

1. BREAKFAST- What is the ratio?
2. MORNING SNACK- Do you use a ratio or fixed/set dose?

- Ratio
- Fixed/Set dose

1. MORNING SNACK- What would be a “typical” dose when your blood sugar is between 4 and 8 mmol/L?
2. MORNING SNACK- How many carbs do you eat (on average)?
3. LUNCH- Do you use a ratio or fixed/set dose?

- Ratio
- Fixed/Set dose

1. AFTERNOON SNACK- Do you use a ratio or fixed/set dose?

- Ratio
- Fixed/Set dose

1. DINNER- Do you use a ratio or fixed/set dose?

- Ratio
- Fixed/Set dose

1. BEDTIME SNACK- Do you use a ratio or fixed/set dose?

- Ratio
- Fixed/Set dose

1. OVERNIGHT- Do you use a ratio or fixed/set dose?

- Ratio
- Fixed/Set dose

1. OTHER- Do you use a ratio or fixed/set dose?

- Ratio
- Fixed/Set dose

**Insulin Delivery**

1. How often do you miss insulin boluses/injections?

- Never
- 1-2 times per month
- Once a week
- 1-2 times per week
- 3 or more times per week

1. At what times of the day are you likely to miss an insulin bolus/injection?

Check all that apply:

- Breakfast
- Between breakfast and lunch
- Lunch
- Between lunch and supper
- Supper
- Before bed
- Overnight
- No particular time

**Blood Sugar Monitoring**

1. On average, how many times per day do you check your blood sugar by finger poke?
2. Do you have Continuous Glucose Monitoring (eg. Dexcom, FreeStyle Libre)?

- **Yes**
- No

1. Which one?

- Dexcom
- Libre
- Medtronic
- Other

1. How much do you use it?

- All or most of the time
- Sometimes
- Rarely or not using

1. Do you have Continuous Glucose Monitoring (eg. Dexcom, FreeStyle Libre)?

- Yes
- **No**

1. Because?

Select all that apply:

- Not interested
- Don’t know about it
- Cost/no insurance
- Tried, but didn’t like it/didn’t work well
- Not on insulin so not needed
- Other

1. How do you review your blood sugars for patterns?

Select all that apply:

- Not reviewed
- See day to day BGs on meter or phone
- Review summary of BGs as needed (using Clarity, LibreView, Glooko, Carelink, meter upload, etc.)
- Routinely review BG summaries (i.e weekly or monthly)
- At clinic visits

1. On average, how often do you review your blood sugars to look for trends and the need for insulin dose adjustment?

- When I notice my blood sugars are high or low
- At least once a week
- At least once a month
- Less than once per month
- At clinic visits

1. Have you made any insulin dose adjustments since the last visit?

- Yes
- No

1. This was done by:

- Child/youth independently
- Parent/caregiver independently
- Child/youth and parent/caregiver together
- Child/youth with assistance from diabetes educators
- Parent/caregiver with assistance from diabetes educators

**High BG and Ketones**

1. Are you checking for ketones when blood sugar is above 17 (or 14 when sick)?

- Always
- Sometimes
- Rarely
- Has not needed to
- Never

1. Have you had positive ketones since the last visit?

- Yes
- No

**Hypoglycemia**

1. How many low blood sugars do you have in a week (on average)?

- 0-2
- 3-4
- 5-6
- Greater than 6

1. What are the usual reasons for your low blood sugars?

Select al that apply:

- Increased activity
- Too much insulin
- Food related (incorrect carb count or not eating everything)
- Other
- None identified

1. Please specifiy:
2. At what time of the day are low blood sugars usually happening?

Select all that apply:

- Breakfast
- AM Snack
- Lunch
- PM Snack
- Supper
- Bedtime
- Overnight
- Variable (no pattern identified)

1. Do you carry fast acting carbs to treat low blood sugar?

- Yes always
- Most of the time
- Sometimes
- No

1. Do you feel your low blood sugars (under 3.5 mmol/L)?

- Yes always
- Most of the time
- Sometimes
- No

1. Do you test blood sugars before treating?

- Yes always
- Most of the time
- Sometimes
- No

1. Have you had a low blood sugar since the last visit that you were too confused or weak to treat without extra help?

- Yes
- No

1. Have you had a severe low blood sugar since the last visit (with seizure or loss of consciousness)?

- Yes (details will be reviewed with your diabetes physician)
- No

1. Have you had to use glucagon since the last visit?

- Yes – Full dose
- Yes- Mini dose
- No

**Nutrition**

1. Do you carb count?

- No
- Sometimes
- Yes

1. How?

Select all that apply:

- Weighing/measuring
- Label reading
- App/online resources
- Estimating

1. On average, approximately how many grams of carbs do you eat per day?

- 0-30g
- 30-60g
- 60-100g
- 100-200g
- 200-300g
- Greater than 300g

1. Do you have a meal plan?

- Yes
- No

1. Do you follow it?

- Yes
- Sometimes
- No

**Physical Activity**

1. What sports or activities do you do and how often?
2. Do you make any adjustments for physical activity ahead of time?

- No
- Sometimes
- Yes

1. Do you:

Select all that apply:

- Decrease insulin
- East extra carbohydrates ahead of time
- N/A

**Other**

1. Do you wear a MedicAlert or have a Medical ID on your phone?

- Yes
- No (has one, not wearing)
- No (does not have one)

1. Have there been any changes at home that you would like us to know about?

- Yes
- No

1. Do you have an up-to-date school plan?

- Yes
- No
- N/A

1. How are the supports at school?

- Good
- Moderate
- Insufficient

1. Are you driving?

- Yes
- No

1. What licence do you have?

- G1
- G2
- G
- Other

**Your Health**

1. Are you experiencing any of the following symptoms?

Select all that apply:

- Fatigue
- Change in appetite
- Abdominal pain
- Diarrhea
- Constipation
- Nausea/vomiting
- Dry hair and/or skin
- Heat or cold intolerance
- Low mood
- Anxiety
- Irregular periods
- Other

1. Other symptoms:
2. Do you have any health insurance?

- Provincial insurance
- Private health insurance
- Don’t know
- None

1. Date of last eye exam (approximately if you’re not exactly sure):
2. That eye exam was:

- Normal
- Abnormal (your physician will review this with you)
