# Supplementary material for: Evaluating Patient-Entered Electronic Health Data as a Strategy to Improve Quality of Care in a Diabetes Clinic: Protocol for a Randomized Controlled Trial
Source: JMIR Res Protoc. 2026 May 8;15:e89519. doi: 10.2196/89519 (PMC13155505; doi:10.2196/89519)
Supplement: Multimedia Appendix 2 [file resprot-v15-e89519-s002.pdf]

Baseline Demographics & Chart Review

Record ID ______________________

Demographic Information

Date of Birth ______________________

Sex
- ◯ Male
- ◯ Female
- ◯ Other

If other, please specify: ______________________

Medical Information

CGM Information

CGM Use?
- ◯ Yes
- ◯ No

Type of CGM
- ◯ Dexcom
- ◯ Libre
- ◯ Medtronic
- ◯ Other

If other, please specify: ______________________

Time CGM active within previous 14 days (%) ______________________

Time in range in last 14 days (%) ______________________

Time below range in previous 14 days (%) ______________________

Time above range in previous 14 days (%) ______________________

Mean glucose (mmol/L) ______________________

Mean glucose standard deviation ______________________

Coefficient of variation ______________________

GMI (%) ______________________
