# Supplementary material for: Evaluating Patient-Entered Electronic Health Data as a Strategy to Improve Quality of Care in a Diabetes Clinic: Protocol for a Randomized Controlled Trial
Source: JMIR Res Protoc. 2026 May 8;15:e89519. doi: 10.2196/89519 (PMC13155505; doi:10.2196/89519)
Supplement: Multimedia Appendix 3 [file resprot-v15-e89519-s003.pdf]

Additional Medical Information Form

Record ID ______________________________

Medical Information

CGM Information

CGM Use?
- ◯ Yes
- ◯ No

Type of CGM
- ◯ Dexcom
- ◯ Libre
- ◯ Medtronic
- ◯ Other

If other, please specify: ______________________________

Frequency of CGM Use
- ◯ All or most of the time
- ◯ Sometimes
- ◯ Rarely or not using

Time CGM active within previous 14 days (%) ______________________________

Time in range in last 14 days (%) ______________________________

Time below range in previous 14 days (%) ______________________________

Time above range in previous 14 days (%) ______________________________

Mean glucose (mmol/L) ______________________________

Mean glucose standard deviation ______________________________

Coefficient of variation ______________________________

Estimated GMI (%) ______________________________
