# Supplementary material for: Evaluating Patient-Entered Electronic Health Data as a Strategy to Improve Quality of Care in a Diabetes Clinic: Protocol for a Randomized Controlled Trial
Source: JMIR Res Protoc. 2026 May 8;15:e89519. doi: 10.2196/89519 (PMC13155505; doi:10.2196/89519)
Supplement: Multimedia Appendix 4 [file resprot-v15-e89519-s004.pdf]

Participant Tool and Workflow Survey

Please complete the survey below.

Thank you!

Who is the parent/caregiver completing this survey?

- ◯ Mother
- ◯ Father
- ◯ Other

If other, please specify:

____

---

1. Were you able to complete the entire MyChart pre-clinic questionnaire?

- ◯ Yes, completely
- ◯ Some, but not all
- ◯ No

If no, why not ?

____

---

2. Who completed the MyChart pre-clinic questionnaire?

- ◯ Parent/caregiver(s)
- ◯ Parent/caregiver(s) with patient
- ◯ Patient alone

---

3. Please let us know what section(s) of the MyChart pre-clinic questionnaire was/were difficult to complete.

- ☐ None
- ☐ Visit Information
- ☐ Blood Sugar Monitoring
- ☐ High BG and Ketones
- ☐ Hypoglycemia
- ☐ Nutrition
- ☐ Physical Activity
- ☐ Other
- ☐ Your Health

---

4. Approximately how long did it take you to complete the most recent MyChart pre-clinic questionnaire?

- ◯ Less than 5 minutes
- ◯ 5-10 minutes
- ◯ 10-15 minutes
- ◯ More than 15 minutes

---

5. What did you think about the length of the MyChart pre-clinic questionnaire?

- ◯ Just right
- ◯ Too long
- ◯ Too short

---

6. You received the MyChart pre-clinic questionnaire 7 days before the appointment. Was that timing appropriate?

- ◯ Yes
- ◯ No, earlier would have been better
- ◯ No, later (closer to appointment date) would have been better

---

7. You received a reminder to complete the MyChart pre-clinic questionnaire one day before the appointment. Was that appropriate?

- ◯ Yes
- ◯ No, earlier would have been better
- ◯ No, later (closer to appointment date) would have been better

---

8. Were there any questions missing from the MyChart pre-clinic questionnaire that you think might have been helpful?

- ◯ Yes
- ◯ No

If yes, what should have been included?

____

9. Were there any questions in the MyChart pre-clinic questionnaire that you feel were not needed?

◯ Yes
◯ No

If yes, which one(s)?

____

10. How did completing the MyChart pre-clinic questionnaire affect the care you received?

◯ It improved the care I received
◯ It did not change the care I received
◯ It worsened the care I received

11. Do you think the care you received during your recent visit was different because the doctor had more information ahead of time?

◯ Yes, the doctor having more information ahead of time made my care better
◯ No, the doctor having more information ahead of time did not change the care I received
◯ No, the doctor having more information ahead of time made my care worse

12. What did you like about the MyChart pre-clinic questionnaire?

☐ Prepared me for the visit
☐ Prepared my doctor for the visit
☐ Saved time during the visit
☐ Helped me remember issues that I wanted to talk about at the visit
☐ Helped me think of new questions for the visit
☐ Made me more likely to attend my visit
☐ None of the above
☐ Other

If other, please specify:

____

13) What did you dislike about the MyChart pre-clinic questionnaire?

____

14) Do you have any other comments about the MyChart pre-clinic questionnaire or how it was used in the diabetes clinic? Please share them here:

____
