# Supplementary material for: Evaluating Patient-Entered Electronic Health Data as a Strategy to Improve Quality of Care in a Diabetes Clinic: Protocol for a Randomized Controlled Trial
Source: JMIR Res Protoc. 2026 May 8;15:e89519. doi: 10.2196/89519 (PMC13155505; doi:10.2196/89519)
Supplement: Multimedia Appendix 5 [file resprot-v15-e89519-s005.pdf]

Study End - Provider Survey

Record ID ______________________

Name of Provider Completing this Survey: ______________________

We've attached the MyChart Questionnaire here. You may refer to this for responding to the next set of questions.

[Attachment: "MyChart Questionnaire.pdf"]

MyChart Questionnaire

	Yes, completely	Mostly	Somewhat	No
1) Overall, do you think your patients (i.e., their parent/caregiver) understood the questions in the MyChart Questionnaire?	○	○	○	○
2) Overall, did your patients (i.e., their parent/caregiver) answer the questions in the MyChart Questionnaire correctly and accurately?	○	○	○	○

3) Were there any questions missing from the MyChart Questionnaire that you think might have been helpful? ______________________

4) Were there any questions in the MyChart Questionnaire that you feel should have been phrased or asked differently? ______________________

5) Were there any questions in the MyChart Questionnaire that you feel were not needed? ______________________

6) Do you have any other comments about the MyChart Questionnaire? ______________________

MyChart Questionnaire Workflow

	Yes	No
7) Do you think that the completion of the MyChart Questionnaire affected your workflow?	○	○

If yes, how so? ______________________

	Yes, it caused me to spend less time documenting.	Yes, it caused me to spend more time documenting.	No, it did not change the time I spent documenting.

8) Overall, did the MyChart Questionnaire affect the time you spent documenting? ○ ○ ○

---

9) What would make the MyChart Questionnaire workflow better?

____
