# Supplementary material for: Evaluating Patient-Entered Electronic Health Data as a Strategy to Improve Quality of Care in a Diabetes Clinic: Protocol for a Randomized Controlled Trial
Source: JMIR Res Protoc. 2026 May 8;15:e89519. doi: 10.2196/89519 (PMC13155505; doi:10.2196/89519)
Supplement: Peer Review Report 1 [file resprot-v15-e89519-s006.doc]

| **Principal Investigator:** | **Ellen Goldbloom** |
| --- | --- |
| **Title:** | **Can We Improve Quality of Care in Diabetes Clinic through Patient-Entered Electronic Health Data?** |
| **Score:** | **4.57** |
|  |  |

**Reviewer 1 Comments**

Summary and Overall Comments

Patient data entry into the EMR irrespective of disorder or clinical context is an important promising area, Dr. Goldbloom has forged role in this area which is garnering national and international attention.

The grant is clearly written, the heavy lifting of preparing the questionnaire has been done.

Team and Metrics

Large team with requisite experience, EDI addressed organically by study entrants.

The statistically significant score difference on PEDQ and/or PQMC scales are well defined outcomes.

The way of the future, t1dm a great test case and the applicant has done a ton of preliminary work in setting up this approach,

**Reviewer 2 Comments**

Summary and Overall Comments

This study provides a massive opportunity to implement and study patient-entered EHR data on the impact of patient-reported experience measures in an outpatient clinic. The knowledge and technical aspects are generalizable to a variety of outpatient and even inpatient settings to improve patient experience and ultimately patient outcomes. The PI is well-positioned nationally and internationally to share the study findings broadly. There appears to be significant collaboration amongst the endocrinology groups across Canada - with the sharing of resources, tools, etc. - developing a multi-site study after this pilot would seemingly be feasible. Additionally, there is opportunity for the data to become part of national/international registries.

Very well written, clear, concise. It does not appear that this has been well-studied previously, particularly in this patient population. Objectives are very clear. Timelines are clear. Budget seems reasonable.

Team and Metrics

There appears to be good representation from early to late-career investigators, eHealth representatives and a methodologist/statistician. Several of the team members have successfully published. EDI was addressed through feedback comments during last review - I am satisfied with the response.

The metrics include: patient-reported experience measures, glycemic control and clinic efficiency. Feedback from prior grant review noted primary outcome PQMC tool had only a 6-point scale was thought insufficiently discriminative. The authors clarified that this tool is actually a 36-point scale and more granularity exists with the second PEQD scale incorporating 100 points. Additionally, their statistical plan includes a sample size calculation that detects a between-group difference of 7 and 11 on the PEQD + PQMC scales. Overall, metrics appear well thought-out and achievable.
